# Profiling Alternative 3′ Untranslated Regions in Sorghum using RNA-seq Data

**DOI:** 10.3389/fgene.2020.556749

**Published:** 2020-10-26

**Authors:** Min Tu, Yin Li

**Affiliations:** Waksman Institute of Microbiology, Rutgers, The State University of New Jersey, Piscataway, NJ, United States

**Keywords:** crop, sorghum, RNA-seq, 3′ untranslated regions, alternative 3′UTR, transcriptome analysis, mRNA N^6^- methyladenosine

## Abstract

*Sorghum* is an important crop widely used for food, feed, and fuel. Transcriptome-wide studies of 3′ untranslated regions (3′UTR) using regular RNA-seq remain scarce in sorghum, while transcriptomes have been characterized extensively using Illumina short-read sequencing platforms for many sorghum varieties under various conditions or developmental contexts. 3′UTR is a critical regulatory component of genes, controlling the translation, transport, and stability of messenger RNAs. In the present study, we profiled the alternative 3′UTRs at the transcriptome level in three genetically related but phenotypically contrasting lines of sorghum: Rio, BTx406, and R9188. A total of 1,197 transcripts with alternative 3′UTRs were detected using RNA-seq data. Their categorization identified 612 high-confidence alternative 3′UTRs. Importantly, the high-confidence alternative 3′UTR genes significantly overlapped with the genesets that are associated with RNA N^6^-methyladenosine (m^6^A) modification, suggesting a clear indication between alternative 3′UTR and m^6^A methylation in sorghum. Moreover, taking advantage of sorghum genetics, we provided evidence of genotype specificity of alternative 3′UTR usage. In summary, our work exemplifies a transcriptome-wide profiling of alternative 3′UTRs using regular RNA-seq data in non-model crops and gains insights into alternative 3′UTRs and their genotype specificity.

## Introduction

*Sorghum bicolor* is a crop globally used for food, fodder, and fuel. The importance of sorghum in agriculture and bioenergy is due to its advantages in diversity, genetics, and genomics (Calvino and Messing, 2012; Boyles et al., 2019; Xie and Xu, 2019). Sorghum cultivars are divided into groups based on their usage: grain sorghum, sweet sorghum, forage sorghum, and energy sorghum. Grain sorghum ranks 5th in global cereal production (Boyles et al., 2019); grain sorghum and forage sorghum serve as significant sources for animal feed; sweet sorghum and energy sorghum are promising bioenergy crops for sugar- and lignocellulosic-based biofuels (Mullet et al., 2014; Mathur et al., 2017; Li et al., 2018; Yang et al., 2020). Sorghum has a relatively small diploid genome with several reference assemblies (Paterson et al., 2009; Deschamps et al., 2018; McCormick et al., 2018; Cooper et al., 2019).

RNA-seq has become a ubiquitous tool in shaping our understanding of the functions of the genomic components in plants (Stark et al., 2019). RNA-seq data are not only valuable in hypothesis-driven research but also powerful for data-driven analysis to generate new insights and testable hypotheses, directing future functional studies. Its primary applications are to measure the gene expression levels and to calculate differential gene expression (DGE). Like in model species and major crops, RNA-seq has recently been widely employed in sorghum research and advanced our understanding in many aspects of sorghum including development (Davidson et al., 2012; Kebrom et al., 2017; Turco et al., 2017; Leiboff and Hake, 2019), accumulation of sugar/biomass (McKinley et al., 2016, 2018; Mizuno et al., 2016, 2018; Zhang L. M. et al., 2018 Li et al., 2019a,b; Zhang et al., 2019; Hennet et al., 2020), stress responses and tolerance (Dugas et al., 2011; Gelli et al., 2014; Sui et al., 2015; Fracasso et al., 2016; Yang et al., 2018; Varoquaux et al., 2019), senescence (Johnson et al., 2015; Wu et al., 2016a), and regulation of miRNA and long non-coding RNA (Calvino et al., 2011; Sun et al., 2020).

3′UTR harbors important regulatory elements, *i.e.*, poly(A) signals and *cis*-acting elements. Poly(A) signals determine polyadenylation (PA), while cis-acting elements in 3′UTR interact with *trans*-acting factors, such as miRNAs and RNA-binding proteins (Ji et al., 2014). 3′UTR-mediated gene regulation affects mRNA localization, translation, transport, and stability (Srivastava et al., 2018). The usage of 3′UTR is complex and regulated. Multiple transcripts can be produced from the same gene through alternative splicing (AS), which could lead to alternative 3′UTRs (Abdel-Ghany et al., 2016). Additionally, a transcript can produce several isoforms with different 3′UTRs varied in length through alternative polyadenylation (APA) (Wu et al., 2011). Alternative 3′UTRs and poly(A) sites are dynamically regulated by developmental and environmental conditions and are tissue specific (Fu et al., 2016; Lin et al., 2017; Lorenzo et al., 2017; Sun et al., 2017; Téllez-Robledo et al., 2019).

To characterize the dynamics of 3′UTRs and poly(A) sites at the genome-wide level, extensive efforts have been made both in experimental and bioinformatic approaches. Several methods based on oligo(dT)-priming have been established to sequence mRNA poly(A) tails, including polyA capture (Mangone et al., 2010), sequencing APA sites (Fu et al., 2011), PolyA Site Sequencing (Shepard et al., 2011), and PA-seq (Hafez et al., 2013). Besides these, other specialized sequencing methods to capture the 3′ ends of mRNAs have been developed, such as poly(A)-position profiling by sequencing (Jan et al., 2010) and 3′ region extraction and deep sequencing (Hoque et al., 2013). In particular, PolyA-Tag sequencing has been extensively used for genome-wide APA studies in higher plants, including *Arabidopsis* (Shen et al., 2011; Wu et al., 2011, 2016b; Hong et al., 2018), *Chlamydomonas reinhardtii* (Bell et al., 2016), rice (Fu et al., 2016; Ye et al., 2019; Zhou et al., 2019), and *Medicago* (Wu et al., 2014). In contrast to the experimental approaches targeting poly(A) tails and/or 3′UTRs, many bioinformatic methods and pipelines have been developed to identify poly(A) sites and/or detect differential APA usage between regular RNA-seq samples [reviewed by Chen et al. (2020)]. These bioinformatics tools fall into four major types: type 1 tools include QAPA (Ha et al., 2018) and PAQR (Gruber et al., 2018) and rely on a pre-existed annotation of poly(A) sites, such as PolyA_DB (Lee et al., 2007), Polysite (Gruber et al., 2016), APASdb (You et al., 2014), and PlantAPA (Wu et al., 2016b); type 2 tools depend on RNA-seq-based transcript reconstruction to infer 3′UTRs, including 3USS (Le Pera et al., 2015) and ExUTR (Huang and Teeling, 2017); type 3 tools, such as Kleat (Birol et al., 2015) and ContextMap 2 (Bonfert and Friedel, 2017), identify poly(A) sites using poly(A)-capped reads in RNA-seq data; and type 4 software include a majority of tools, namely, PHMM (Lu and Bushel, 2013), GETUTR (Kim et al., 2015), Change-Point (Wang W. et al., 2014), EBChangePoint (Zhang and Wei, 2016), IsoSCM (Shenker et al., 2015), DaPars (Xia et al., 2014), APAtrap (Ye et al., 2018), and TAPAS (Arefeen et al., 2018). The type 4 methods identify APA dynamics based on modeling read density fluctuation in RNA-seq data. The advantages and the limitations for each type of the bioinformatic tools have been reviewed in detail, and their performance was compared using benchmarking datasets (Chen et al., 2020).

Previous studies focused on the experimental methods for sequencing poly(A) tails have enhanced our understanding of the functions of the core components of poly(A) machinery, cleavage and polyadenylation specificity factor complex (F), including CPSF30, CPSF100, and FIP1. CPSF30 affects poly(A) signal recognition on the near-upstream element and thus the choice of poly(A) sites for many genes in *Arabidopsis* (Thomas et al., 2012; Chakrabarti and Hunt, 2015). CPSF100 affects the poly(A) site choices through the far-upstream element, resulting in transcriptional read-through for many genes (Lin et al., 2017). Another subunit called FIP1 (factor interacting with PAP1), which acts as a bridge between poly(A) polymerase (PAP) and CPSF (Preker et al., 1995), has complex roles in regulating poly(A) site selection and responses to ABA and abiotic stresses in *Arabidopsis* (Téllez-Robledo et al., 2019). While these studies immensely contribute to understanding the dynamics, mechanisms, and variations in plant polyadenylation sites, these methods require specialized library-prep protocols and bioinformatic pipelines. Unlike routine RNA-seq, they are technically challenging and more expensive, preventing from becoming widely available for plant scientists. Many plant scientists work on agriculturally and economically important crops with large and complex genomes [*e.g.*, maize (Jiao et al., 2017), sorghum (Paterson et al., 2009; McCormick et al., 2018), wheat (International Wheat Genome Sequencing Consortium [IWGSC], 2008), and sugarcane (Zhang J. et al., 2018)], which present potential technical difficulties when performing multi-omics experiments. Although sequencing costs are rapidly decreasing, the majority of published RNA-seq data available in plants, especially those in non-model crops, were produced using the Illumina short-read sequencing platforms (Stark et al., 2019). For example, in sorghum, a large amount of RNA-seq studies were conventional short-read sequencing used for DGE analysis, with a few analyzing AS (Abdel-Ghany et al., 2016). These situations suggest that 3′UTR analysis using conventional RNA-seq data should be valuable and significant for non-model plants.

In this study, we sought to gain insights into alternative 3′UTRs in sorghum. Due to the importance of sorghum stem as a carbon reservoir and a conduit for the mobilization of water, nutrients, and signaling molecules, we chose to analyze the RNA-seq data of sorghum stems, which was previously generated by our group in three genetically related but phenotypically contrasting lines—Rio, BTx406, and R9188—to identify genes and regulatory networks associated with stem sugar accumulation in sweet sorghum (Li et al., 2019a,b). Our present study has the following objectives: (1) to characterize alternative 3′UTRs in sorghum stems, (2) to gain insights into 3′UTR regulation, and (3) with the identified alternative 3′UTRs, to generate testable hypotheses for future in-depth functional studies of 3′UTR-mediated regulation in sorghum.

## Materials and Methods

### RNA-seq for Profiling 3′ Untranslated Regions

The RNA-seq dataset (NCBI accession PRJNA413691) used for 3′UTR analysis was generated previously by us to study genes and networks associated with soluble sugar accumulation in internodes (internodes 2, 3, and 4, numbered from top to bottom) at four post-stem elongation stages from three sorghum genotypes, Rio, BTx406, and R9188 (Li et al., 2019a). The three genotypes contrast in the phenotypes of stem sugar accumulation: Rio accumulates high contents of sugar in stems during the post-flowering stages (∼20% Brix in the stem-extracted juice), while BTx406 has a low stem sugar content (<10% Brix), with R9188 having an intermediate stem sugar content especially during the post-flowering stages (Li et al., 2019a). R9188 is an introgression line developed from the BTx406/Rio cross followed by one backcross to sweet sorghum Rio and contains the early flowering and dwarf loci introgressed from grain sorghum BTx406 (Ritter et al., 2004). The dataset has an advantage such that the three genetically related genotypes allow us to investigate inter-genotype 3′UTR variations.

For each genotype, the internode samples were collected at four time points, namely, T1, T2, T3, and T4 (flag leaf stage, 100% flowering, 10 days after flowering, and 15 days after flowering, respectively), in the experimental field under a split plot design. RNA was extracted with the TRIzol method, processed to libraries, and sequenced using 150-bp pair-end according to standard protocols. Regular quality control and filtering steps were applied to the raw reads and then mapped to the sorghum reference genome BTx623 (v2.1) with Tophat (Paterson et al., 2009; Trapnell et al., 2012). To calculate gene expression (in reads per kilobase of exon per million mapped sequence reads, RPKM) and differentially expressed genes (DEGs), uniquely mapped reads were used, and DEGs were determined with DEseq and edgeR (Anders and Huber, 2010; McCarthy et al., 2012). Moreover, 18,275, 19,727, and 19,102 genes were expressed in Rio, BTx406, and R9188, respectively (total number of the expressed genes for all timepoints, genic reads per replicate ≥ 10, average RPKM per sample ≥ 1).

### Transcriptome-Wide Analysis of Alternative 3′UTRs

The publicly available bioinformatic method priUTR (*P*rogram for *R*NA-seq-based *I*dentification of Alternative 3′*UTR*) was used in this study to identify alternative 3′UTRs using regular RNA-seq data from sorghum (Tu, 2020). The program and its user manual are available at https://github.com/mint1234/3UTR-. priUTR is a type-2 bioinformatic method for identifying alternative 3′UTRs, which relies on RNA-seq-based transcript reconstruction to infer mRNA 3′ end. Detailed information about this method is provided in Supplementary Figure S1. The alternative 3′UTR results for each genotype and timepoint were obtained with priUTR after mapping the sorghum RNA-seq data to the BTx623 reference genome using a reference-guided mode with Tophat. The reference-guided transcriptome assembly (in GTF format) and gene expression data generated from the Tophat-Cufflinks pipeline were used as input files for the priUTR program. Only those alternative 3′UTRs detected in all three replicates per genotype and time point were kept. Hereafter, a gene or a transcript with an alternative 3′UTR is mentioned as an alternative 3′UTR gene or an alternative 3′UTR transcript.

We integrated the BTx406-introgressed genes/alleles to find out alternative 3′UTR genes associated with the genetic relationship among R9188, Rio, and BTx406 (Li et al., 2019a). A total of 1,805 genes introgressed from BTx406 into R9188 were previously identified based on RNA-seq-derived SNPs with SAMTools, followed by a series of stringent filters, including the mapping quality, read depth, bi-allelism, and homogeneity and homozygosity of the SNPs. The RNA-seq read mapping data were examined for the alternative 3′UTR genes and compared between Rio, BTx406, and R9188 using a genome browser to identify the alternative 3′UTRs that were specifically detected in Rio but not in BTx406/R9188 or *vice versa*.

### Expression of Sorghum m^6^A Functional Factors

The functional factors (“reader,” “writer,” and “eraser” proteins) of m^6^A modifications in *Arabidopsis* were reported previously (Duan et al., 2017; Ruzicka et al., 2017; Arribas-Hernández et al., 2018; Scutenaire et al., 2018; Wei et al., 2018), with their maize homologs phylogenetically identified (Miao et al., 2020). The protein sequences of the m^6^A functional factors were used for a BLAST search in the sorghum genome, with a threshold of E value ≤ 10^–5^. The best BLAST hits in sorghum were also verified by the gene orthology between maize and sorghum (Zhang et al., 2017).

### Functional Annotation and Enrichment Analysis

To comprehensively understand the functions of alternative 3′UTR genes, the functional annotation containing three major sources of annotation, Gene Ontology (GO), MapMan, and KEGG, was employed (Li et al., 2019a). The GO annotation was from Phytozome and AgriGO (Du et al., 2010). For MapMan, the second-level bincodes of the annotation was used (Usadel et al., 2009). Enriched functional terms of the high-confidence alternative 3′UTR geneset (group 4, see “RESULTS”) or the m^6^A associated geneset were calculated using clusterProfiler (*hypergeometric* < 0.05; Yu et al., 2012).

## Results

### Transcriptome-Wide Identification of Alternative 3′UTRs in Sorghum Using RNA-seq Data

**FIGURE 1:**
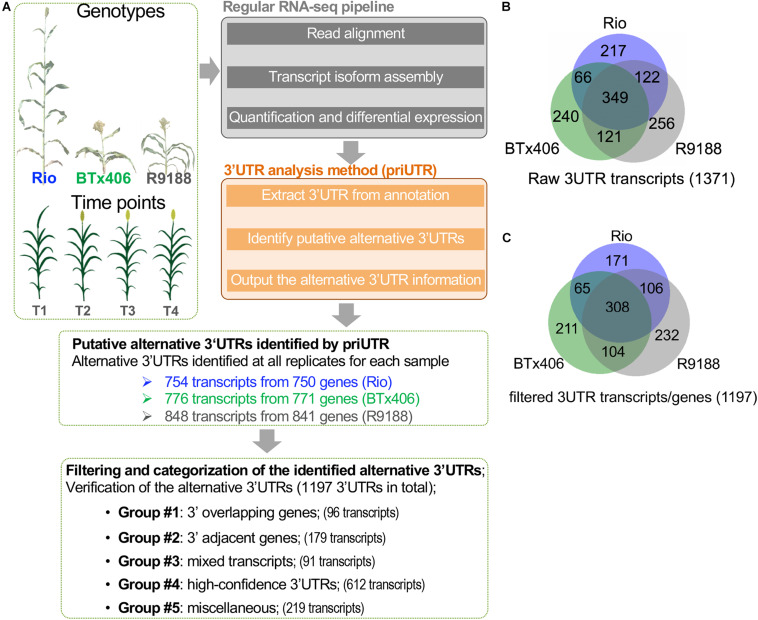
Characterization of the alternative 3′ untranslated regions (3′UTRs) in sorghum internodes. **(A)** A diagram showing the sorghum 3′UTR analysis workflow using the published RNA-seq datasets of sorghum internodes at four post-elongation time points. The RNA-seq datasets were from an introgression line R9188 and its two parental lines Rio and BTx406, with contrasting phenotypes in plant height, flowering time, and stem sugar accumulation. 3′UTR analysis was performed using the publicly available 3′UTR tool (priUTR) to identify putative alternative 3′UTRs compared to the sorghum reference genome (cultivar BTx623, v2.1). A total of 1,371 predicted alternative 3′UTRs transcripts were initially identified, with 754, 776, and 848 transcripts detected in Rio, BTx406, and R9188, respectively. Further filtering steps refined to 1,197 transcripts, with at least 50-bp 3′UTR differences in length between the annotation and prediction. The 1,197 alternative 3′UTRs were categorized into five groups, leading to the identification of 612 high-confidence alternative 3′UTRs (designated as group 4). The other groups may possibly be false predicted due to several interfering factors, such as 3′ overlapping genes (designated as group 1), 3′ adjacent genes (designated as group 2), and mixed transcripts from the same gene (designated as group 3) (see details in “MATERIALS AND METHODS” and Figure 2). **(B)** Venn diagram of the 1,371 raw alternative 3′UTR transcripts detected in Rio, BTx406, and R9188. **(C)** Venn diagram of the 1,197 alternative 3′UTR transcripts detected in Rio, BTx406, and R9188, with more than 50-bp length difference compared to the corresponding 3′UTRs in the annotation.

**FIGURE 2:**
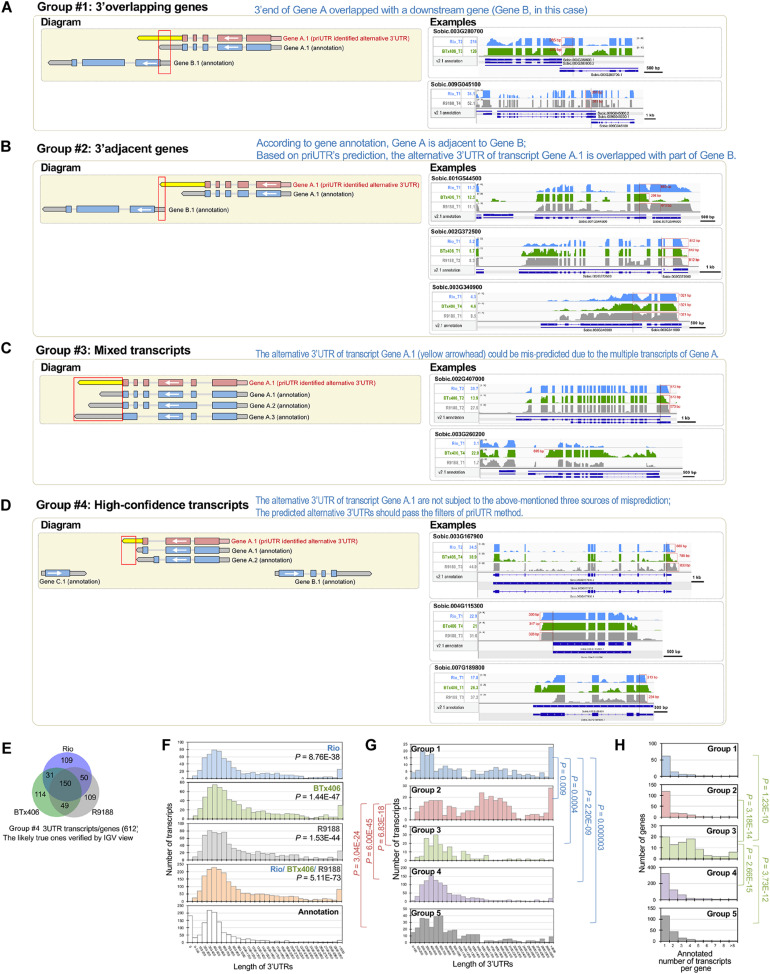
Categorization of the predicted 3′UTRs highlights the high-confidence group of alternative 3′UTRs in sorghum. **(A**–**D)** Schematic diagrams showing that the predicted 3′UTRs in sorghum were categorized into groups, with groups 1, 2, 3, and 4, respectively. In the left panel of the diagrams, the white arrow indicates gene orientation. The red gene model denotes the reconstructed transcript, while the blue gene model denotes the annotated transcript, with the yellow arrow denoting the identified alternative 3′UTR. In the right panel, representative Integrative Genomics Viewer (IGV) views of the predicted alternative 3′UTRs in groups 1, 2, 3, and 4 (**A**–**D**, respectively) are shown. The select IGV tracks of the genotype and time point are shown, in which the alternative 3′UTRs were detected. The gene expression levels at those samples provided (in reads per kilobase of exon per million mapped sequence reads). The alternative 3′UTRs are highlighted in red boxes, with their length labeled. **(E)** Venn diagram of the 612 high-confidence alternative 3′UTR transcripts. **(F)** Histograms showing the length of the high-confidence alternative 3′UTRs detected in Rio, BTx406, and R9188, all of the three genotypes and their corresponding annotations. The results indicated that the identified alternative 3′UTRs tend to be longer than the annotations (Wilcoxon rank sum test, *P* < 0.05). **(G)** The histograms of the alternative 3′UTR length of the five groups demonstrated that groups 1 and 2 differed significantly in 3′UTR length from the other groups (Wilcoxon rank sum test, *P* < 0.05), while the length distribution of 3′UTRs was not different among groups 3, 4, and 5. Longer 3′UTRs in groups 1 and 2 are consistent with the classification that the alternative 3′UTRs in groups 1 and 2 could be mostly mis-detected due to the 3′ downstream genes overlapping with or adjacent to the 3′ ends of the alternative 3′UTR genes, respectively. **(H)** Histograms of the number of transcripts per gene for the five groups showed that group 3 has significantly more transcripts per gene than the other groups (Wilcoxon rank sum test, *P* < 0.05), matching the likely source of false positive for group 3 that the alternative 3′UTRs could be mis-predicted by mixing multiple 3′UTRs of the transcripts from the same gene.

We performed a transcriptome-wide analysis of alternative 3′UTRs using the sorghum internode RNA-seq datasets (Li et al., 2019a). A total of 1,371 transcripts were identified to have alternative 3′UTRs for all the three genotypes. Among them, 754, 776, and 848 transcripts derived from 750, 771, and 841 genes were identified in Rio, BTx406, and R9188, respectively (Figures 1A,B). We focus on analyzing the features of the alternative 3′UTR genes in terms of their 3′UTR lengths, gene order, and functions rather than the 3′UTR dynamics over the time points. Therefore, the alternative 3′UTR transcripts from the four time points were pooled together for downstream analysis. The read depth within a mRNA tends to drop rapidly when reaching to its 3′ end in RNA-seq data, which is known as the 3′ bias of RNA-seq (Li et al., 2015). Due to the 3′ bias of RNA-seq, we removed any transcripts of which the predicted alternative 3′UTR differed from its corresponding annotation, with no more than 50 bp in length to avoid potential bioinformatic artifacts. After that, 1,197 alternative 3′UTRs were kept. Because priUTR is a type-2 bioinformatic tool for alternative 3′UTR detection and could have inherent limitations of transcript assembly tools, the read mapping results of these 1,197 alternative 3′UTRs were visualized on Integrative Genomics Viewer (IGV; Thorvaldsdóttir et al., 2013) and manually compared with their corresponding annotations in BTx623 reference genome in order to find out potential sources of false prediction and to identify high-confidence alternative 3′UTR (Figures 1A,C). Based on the types of possible mis-prediction, the 1,197 transcripts were categorized into five groups (Figure 1A). Group 1 (namely, the “3′ overlapping gene” group) represents the alternative 3′UTRs that could be likely mis-predicted because a gene is located at the 3′ downstream of the alternative 3′UTR gene and overlaps with the predicted 3′UTR (Figure 2A). Group 2 (namely, the “3′ adjacent gene” group) represents the alternative 3′UTRs that could be mis-predicted because a gene locates at the 3′ downstream of the alternative 3′UTR gene and overlaps only with the predicted 3′UTR extension but not with the annotated 3′UTR region (Figure 2B). Group 3 (namely, the “mixed transcript” group) stands for those potentially false predictions, likely due to gene models producing multiple transcripts. In such a situation, the alternative 3′UTR transcript could be embedded within a longer transcript or be mis-predicted due to other transcripts with a similar 3′UTR length (Figure 2C). Group 4 is a collection of high-confidence alternative 3′UTR transcripts of which the results in IGV supported the predicted alternative 3′UTRs (Figures 2D,E). Group 5 are the transcripts of which the alternative 3′UTR seems to be mis-predicted by other miscellaneous reasons. Groups 1, 2, 3, 4, and 5 contains 96, 179, 91, 612, and 219 transcripts, respectively (Figure 1A). Examples of the genome browser views for groups 1, 2, 3, and 4 are shown in Figures 2A–D, respectively.

A comparison of the alternative 3′UTR lengths from three sorghum genotypes with those of the annotation showed significantly longer 3′UTRs in our results (Figure 2F). Moreover, a comparison of the alternative 3′UTR lengths between the five groups indicated that groups 1 and 2 have longer alternative 3′UTRs than the other groups, matching with our findings that many of them are likely false prediction due to the inclusion of transcribed neighboring genes (Figure 2G). Besides that, the “mixed transcript” group (group 3) has significantly higher numbers of transcripts per gene (based on the genome annotation) when compared with the remaining four groups, in agreement with the possible source of false prediction as “mixed transcripts” (Figure 2H). Additionally, a comparison of the expression levels between the five groups showed that the expression levels of the “3′ overlapping” genes (group 1), but not other groups, were lower than those of the high-confidence alternative 3′UTRs (group 4), suggesting that high expression levels may not be a major reason for the high-confidence prediction of alternative 3′UTRs (Supplementary Figure S3). This manual verification step highlights that the high-confidence alternative 3′UTRs consist of a large fraction of all predictions (group 4, 612 out of 1,197) and identifies several major sources of false prediction.

### Association Between Alternative 3′UTRs and RNA m^6^A Modification

Since APA and RNA m^6^A modification are two molecular phenomena that could be associated with changes in 3′UTR in plants, we therefore sought to test whether the alternative 3′UTRs identified here are associated with APA or RNA m^6^A modification.

**FIGURE 3:**
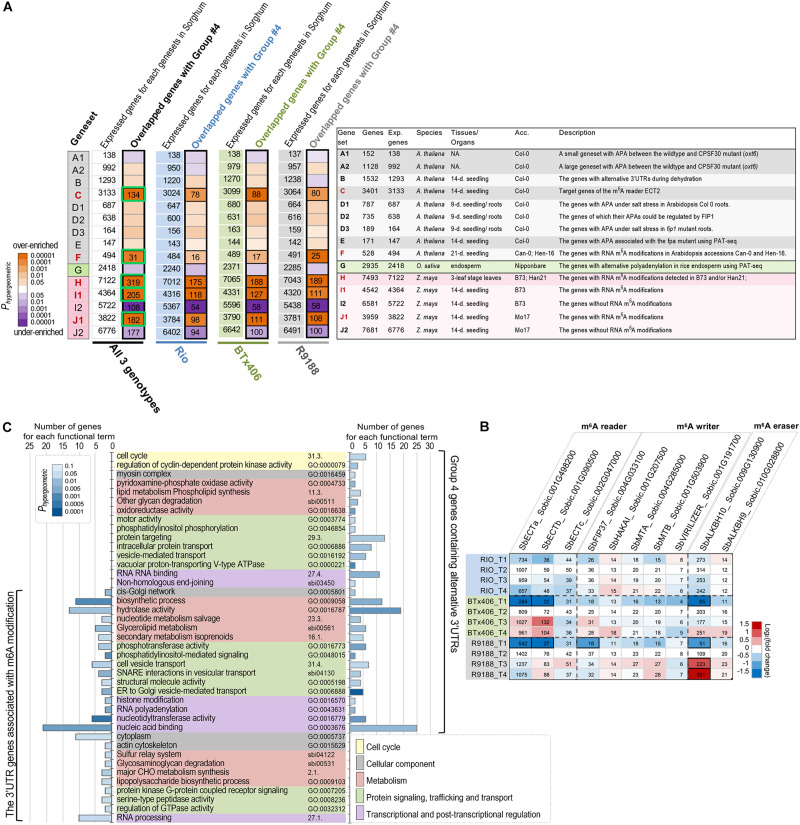
The alternative 3′UTR genes in sorghum are associated with m^6^A modification. **(A)** Significant overlapping between the high-confidence alternative 3′UTR genes in sorghum and the APA- or m^6^A- associated genesets (*P* < 0.01, hypergeometric test). The significant overlaps between genesets are labeled in black boxes, and the number of overlapping genes is shown. Green box highlights group 4 alternative 3′UTR genes potentially associated with m^6^A modifications, which is used for functional enrichment analysis **(C)**. The right panel is a summary list of the published genesets of alternative polyadenylation, alternative 3′UTR, or RNA m^6^A modifications in *Arabidopsis*, rice, and maize. The number of sorghum genes (column “Genes”), expressed genes in sorghum (column “Exp. Genes”) orthologous to those in *Arabidopsis*, rice, or maize, and the original tissues/organs and accessions (column “Acc.”) are given (details of these genesets are provided in Supplementary Table S2). The backgrounds of the genesets are colored according to the original species, and the m^6^A-associated genesets (C, H, I1, and J1) are highlighted in red. The genesets and their corresponding references are as follow: genesets A1/A2—Thomas et al., 2012; geneset B—Sun et al., 2017; geneset C—Wei et al., 2018; genesets D1/D2/D3—Téllez-Robledo et al., 2019; geneset E - Duc et al., 2013; geneset F—Luo et al., 2014; genesets G—Fu et al., 2016; geneset H—Miao et al., 2020; genesets I1/I2/J1/J2—Luo et al., 2020. NA., not available; 9-days, 9-day old; 14-days, 14-day old; 21-days, 21-day old). **(B)** Expression dynamics of the sorghum genes encoding m^6^A readers, writers, and erasers. The heatmap is shaded to reflect the magnitude of log2 fold change of gene expression relative to the T2 timepoint per each genotype. Gene expression values in reads per kilobase of exon per million mapped sequence reads are given. The differentially expressed genes per genotype are highlighted in black boxes. **(C)** Functional enrichment results with GO, MAPMAN, and KEGG terms show that both high-confidence alternative 3′UTR genes (group 4) and its subset m^6^A-associated genes cover a diverse range of function aspects. Only the significant terms (*P* < 0.05, hypergeometric test) with at least two hit genes per term are shown.

APA is one important approach currently known to produce length variations in 3′UTR in plants. Alternative polyadenylation and/or 3′UTRs are regulated by genetic factors, environmental conditions, and developmental context. Polyadenylation (PA) is directly controlled by the CPSF complex that contains six subunits, CPSF30, CPSF73, CPSF100, CPSF160, Wdr33, and FIP1, and recognizes the polyadenylation sites on 3′UTR (Mandel et al., 2008; Shi et al., 2009). Some subunits of the PA machinery (CPSF30, CPSF100, and FIP1) have been functionally characterized in plants (Thomas et al., 2012; Lin et al., 2017; Téllez-Robledo et al., 2019). FPA is another *trans*-acting factor regulating the 3′ end formation of mRNA in *Arabidopsis* (Duc et al., 2013). Besides that, abiotic stresses, such as dehydration and salt, can also induce alternative polyadenylation (Sun et al., 2017; Téllez-Robledo et al., 2019). Recently, a comprehensive profiling of APAs in rice across several developmental stages and tissues marks tissue specificity in APA patterns (Fu et al., 2016). Based on the abovementioned observation, it can be hypothesized that, if the 612 high-confidence alternative 3′UTR transcripts (group 4) use their alternative 3′UTRs are due, at least partly, to the previously studied PA machinery proteins or environmental conditions, a significant fraction of the 612 sorghum transcripts would be the orthologs of the APA genes in *Arabidopsis* or rice which are associated with PA proteins or stresses. To test the APA hypothesis, we retrieved the APA-associated genesets (genesets A1, A2, B, D1, D2, D3, E, and G) from the above-mentioned studies in *Arabidopsis* and rice. The details of these genesets are provided in Figure 3A and Supplementary Tables S2, S3. The gene IDs in *Arabidopsis* and rice were converted to sorghum gene IDs using the established gene orthologous relationships between *Arabidopsis* and sorghum (Van Bel et al., 2018) and those between rice and sorghum (Sakai et al., 2013), respectively. Hypergeometric tests failed to reveal any significant enrichment of the APA-associated genesets within the five groups of sorghum alternative 3′UTR genes (*P* < 0.05; Figure 3A and Supplementary Figure S4), not supporting the APA hypothesis that the alternative 3′UTR genes observed here are largely contributed by the known polyadenylation machinery proteins or the stresses.

We then analyzed whether the alternative 3′UTRs identified in sorghum could be related to RNA m^6^A modification. RNA m^6^A modification is another factor known to be related to 3′UTR, as a large portion of m^6^A sites in plants are located within mRNA 3′UTR regions (Luo et al., 2014, 2020). m^6^A is installed by m^6^A methyltransferase [known as “m^6^A writer,” *e.g.*, methyltransferase-like 3 (METTL3), METTL14, Wilms tumor 1-associated protein (WTAP); Liu et al., 2014; Wang Y. et al., 2014; Ping et al., 2014], recognized by “m^6^A reader” (EVOLUTIONARILY CONSERVED C-TERMINAL REGION proteins, ECT2, 3, 4 in plants; Wei et al., 2018) and removed by “eraser” m^6^A RNA demethylase [*e*.*g*., alkylated DNA repair protein AlkB homolog 5 (ALKBH5); Zheng et al., 2013]. Previously, mRNA m^6^A methylome studies in *Arabidopsis* (Luo et al., 2014), rice (Zhang et al., 2019), and maize (Luo et al., 2020; Miao et al., 2020) reveal a functional association of m^6^A with chloroplast, sporogenesis, stress response, and translational status, highlighting new functions of m^6^A functional factors, the effects of m^6^A modification, and m^6^A inter-variety variation. In the present study, if the 612 alternative 3′UTRs in sorghum are associated with or regulated by m^6^A modification, a significant fraction of the 612 transcripts would be orthologs of the genes with m^6^A modifications in *Arabidopsis* or maize (designated as the m^6^A hypothesis). To investigate the hypothesis, several m^6^A-associated genesets were obtained from the m^6^A-modified genes reported in *Arabidopsis* (genesets C and F) and maize (geneset H, I1, and J1; Figure 3A; Luo et al., 2014, 2020; Miao et al., 2020). The genesets I2 and J2 exclusively contain the expressed genes without m6A modification in maize seedlings, serving as the controls for genesets I1 and J1, respectively (Luo et al., 2020). Our analysis clearly showed that the high-confidence alternative 3′UTR genes (group 4) are significantly over-enriched with the orthologs of m^6^A-associated genesets in both *Arabidopsis* and maize but are under-enriched with the m^6^A control genesets (I2 and J2) (*P_*hyp*__*er*__*geometric*_* < 0.01; Figure 3A). For example, many genes which are orthologous targets of m^6^A reader ECT2 in *Arabidopsis* were significantly over-enriched in group 4. The hypergeometric tests were first performed using all of the 612 alternative 3′UTR genes pooled from three sorghum genotypes. We further repeated the analysis in each of the three sorghum lines to avoid potential statistical artifacts. The results for each sorghum genotype confirmed the over-enrichment of m^6^A-associated genes (Figure 3A and Supplementary Tables S4, S5). The statistical significance of the enrichment was further assessed using 400 permutation tests for each of the three sorghum genotypes. In group 4, there are 340, 344, and 358 genes from Rio, BTx406, and R9188, respectively. To avoid potential influences of expression levels in the permutation tests, we examined the distribution of expression levels of the 612 alternative 3′UTR genes within each sorghum genotype (Supplementary Table S5). The deciles of the expression levels of group 4 genes were determined in Rio, BTx406, and R9188, respectively, and used to randomly select 36 expressed genes within each expression decile per genotype from all of the expressed genes, yielding a set of 360 random genes with a distribution of expression levels similar to that of the group 4 genes. The results of permutation tests showed that both the number of overlapped genes and the hypergeometric *p*-values were significantly correlated with m^6^A genesets C, F, H, I1, and J1 (Supplementary Figures S5, S6). In contrast, the number of overlapped genes and the hypergeometric *p*-values were significantly smaller than the random distributions for m^6^A control genesets I2 and J2 (Supplementary Figures S5, S6), supporting a significant under-enrichment of non-m^6^A modified genes in the group 4 genes. Overall, the analysis between genesets is suggestive of the m^6^A hypothesis.

To ascertain m^6^A occurrence in sorghum internodes, we found that multiple genes putatively encoding m^6^A reader, writer, and eraser, respectively, were expressed in the RNA-seq samples. with many of them expressed at high levels. The genes encoding m^6^A readers (Sobic.001G498200 and Sobic,001G090500) and eraser (Sobic.009G130900) were upregulated during post-anthesis stages in BTx406 and R9188 (Figure 3B).

To portrait the representative molecular functions, biological processes, and metabolic pathways, functional enrichment was performed for the alternative 3′UTR genes and the subset of 3′UTR genes associated with m^6^A modifications. The subset of m^6^A-associated 3′UTR genes was generated by combining the overlapping genes across several m^6^A genesets (green boxes in Figure 3A, a total of 398 genes). Figure 3C reveals that both the alternative 3′UTR genes and the m^6^A-associated 3′UTR genes cover a diverse set of functions, which can be grouped into five major categories, including cell cycle, cellular components, metabolism, protein signaling, trafficking and transport, and transcriptional and post-transcriptional regulation. The enrichment results of these two genesets highlight functions related to metabolism (*i*.*e*., biosynthetic process, hydrolase activity) and nucleic acid regulation (*i*.*e*., nucleic acid binding and RNA processing). Moreover, we found that the sorghum m^6^A-associated 3′UTR genes are highly enriched in RNA processing, nucleic acid binding, as well as functions associated with protein localization and transportation (for example, cell vesicle transport, SNARE in vesicle transport, *cis*-Golgi network) (Figure 3C), consistent with the representative functions of m^6^A-modified genes previously seen in *Arabidopsis* and maize (Luo et al., 2014, 2020).

### Genotype-Specific Alternative 3′UTRs

**FIGURE 4:**
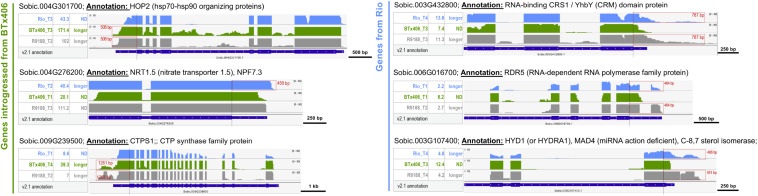
The alternative 3′UTRs in sorghum can be genotype specific. Representative examples of genotype-specific alternative 3′UTRs. Based on the SNPs identified using RNA-seq reads from Rio, BTx406, and R9188, the genes in R9188 were identified to be originated from Rio or introgressed from BTx406. Examples of alternative 3′UTRs distinct between Rio and BTx406/R9188 (the R9188 alleles also introgressed from BTx406): a longer 3′UTR of Sobic.004G276200 was detected only in Rio but not in BTx406/R9188, while longer 3′UTRs of Sobic.004G301700 and Sobic.009G239500 were seen only in BTx406/R9188 but not in Rio. Examples of alternative 3′UTRs distinct between Rio/R9188 and BTx406 (the R9188 alleles originated from Rio): longer 3′UTRs of Sobic.003G432800, Sobic.006G016700, and Sobic.003G107400 were detected in Rio/R9188 but not in BTx406. The select Integrative Genomics Viewer tracks of the genotype and time point are shown, in which the alternative 3′UTRs were detected. The gene expression levels of those samples are provided (in reads per kilobase of exon per million mapped sequence reads). The alternative 3′UTRs are highlighted in red boxes, with their length labeled. ND, not detected.

Utilizing the genetic relationship among Rio, BTx406, and R9188, we sought to address whether alternative 3′UTR can be genotype-specific. The RNA-seq read mapping data of the 612 alternative 3′UTRs were compared between the three genotypes. Twenty-three genes were identified to have distinct alternative 3′UTRs either between Rio and BTx406/R9188 (seven genes, Supplementary Figure S7A) or between Rio/R9188 and BTx406 (16 genes, Supplementary Figures S7B,C). Many of them (19 out of 23) are homologous to the genes with known annotations or proven important functions in *Arabidopsis*. The genotype-specific alternative 3′UTRs comprise only a small fraction of the identified alternative 3′UTRs (∼3.7%, 23 out of 612). To further validate the parental origins of these 23 alternative 3′UTR genes in R9188, we utilized the 1,805 genes that are introgressed from BTx406 into R9188 (Li et al., 2019a). All of the seven genes in R9188 with identical 3′UTR length to BTx406 are indeed introgressed from BTx406, while among the 16 R9188 genes with identical 3′UTR length to Rio, eight are validated to be originated from Rio, with the remaining eight genes lacking SNPs for validation (Supplementary Figure S7). The representative genes in R9188 that have genotype-specific alternative 3′UTRs are shown in Figure 4. Many homologs of the genes identified here are critical for plant development. For example, Sobic.004G276200 is a homolog of AtNRT1.5 (AT1G32450) that regulates root architecture and leaf senescence through nitrate response and root-to-shoot nitrate transport and potassium translocation (Meng et al., 2015; Zheng et al., 2016). Sobic.006G016700 is homologous to AtHYD1 (AT1G20050), a sterol biosynthetic gene that affects cell wall synthesis, phytohormone signaling, and miRNA activity, crucial for embryo and root development (Schrick et al., 2004; Souter et al., 2004; Brodersen et al., 2012).

## Discussion

Our work exemplifies a transcriptome-wide 3′UTR analysis using conventional RNA-seq data in non-model plants, identifying several hundred genes with high-confidence alternative 3′UTRs in sorghum. While many bioinformatic tools for identifying mRNA 3′ ends have been reported, the priUTR program was used here to analyze sorghum 3′UTRs for several reasons (Tu, 2020). First, the genome assembly and annotation files of non-model crop species, such as maize and sorghum, can be readily used as input files of priUTR (*e*.*g*., gff3 annotation), while some other programs appear to lack clear guidelines for preparing input files using non-model plant species. Second, results from the priUTR tool should be considered largely reliable, since priUTR resembles a previous online tool for detecting alternative 3′UTRs, 3USS, in the principles of the algorithms (Le Pera et al., 2015). priUTR adopts the idea of intron-chain matching from 3USS, which required the transcript reconstruction from Cufflinks or Scripture as one of the input files and predicted alternative 3′UTRs once all of the intron chain could be matched between the assembled and annotated transcript. However, 3USS provided alternative 3′UTR prediction for model animals and *Arabidopsis* and is no longer operational. Unlike 3USS, priUTR utilizes partial intron-chain matching algorithm, in which three consecutive introns before the stop codon are required to be matched and holds the flexibility that the assembled transcript could have alternative splicing in its 5′ part or have additional unannotated introns in the predicted 3′UTR regions (Supplementary Figure S1). Third, the priUTR tool could be used for analyzing multiple RNA-seq samples by program loop or parallelism. Fourth, the priUTR program fits our computer systems (Scientific Linux release 6.10 Carbon), while other programs might not be readily compatible with our operational system.

Extensive bioinformatics efforts have been made to identify poly(A) sites or mRNA 3′ tails using RNA-seq data. These previous bioinformatic tools have been classified into four groups based on their principles of the algorithms (Chen et al., 2020). Some of the programs have been compared using benchmarking datasets from model species (*i.e.*, human, mouse, and *Arabidopsis*). The advantages and the limitations for each type of poly(A)-site prediction tools have been well discussed. Type 1 tools depend on a prior database of poly(A) sites in model animals or *Arabidopsis* and can effectively identify numerous poly(A) sites. Type 1 tools are limited to detect previously known poly(A) sites. Type 2 programs apply relatively mature software for transcriptome assembly, such as Scripture (Guttman et al., 2010), Cufflinks (Trapnell et al., 2012), and StringTie (Pertea et al., 2016). While type 2 programs could detect both 3′UTR extending and shortening events, they inherit the limitations of transcript assembly tools that are: (1) failure to distinguish the transcripts which overlap or reside closely between each other in the genome and (2) inability to distinguish a short transcript isoform embedded in longer ones from the same gene. Type 3 tools heavily depend on poly(A)-capped reads, which are scarce in regular RNA-seq data (Bayerlova et al., 2015; Kim et al., 2015), leading to low sensitivity of these methods. Type 4 programs detect sudden fluctuations of read density at the 3′ end of mRNAs in order to model 3′UTR usage. Type 4 programs tend to identify 3′UTR shortening events and have more inaccurate predictions caused by the heterogeneity of read coverage and non-biological variations (Szkop and Nobeli, 2017). Based on the information above, the type 2 methods which rely on transcript reconstruction and infer 3′UTR from assembled transcripts may be a potentially better choice for alternative 3′UTR study for non-model plant species with reference genome available.

As a type 2 bioinformatic tool for alternative 3′UTR prediction, priUTR has some limitations. It heavily relies on the accuracy of transcript annotation, and the accuracy of RNA-seq-based transcript reconstruction depends on the genic context. When multiple transcripts are produced from a gene model, of which the transcript with a shorter 3′UTR sequence embedded within a longer transcript, transcript reconstructions are prone to be inaccurate. Such cases have been identified in our study as the group 3 alternative 3′UTRs (Figure 2). In other situations, when several gene models are located closely in the same genomic locus, the prediction of 3′UTR extension tends to be false because the transcript could be mis-assembled to include neighboring genes. In this study, these likely mis-predicted alternative 3′UTRs were categorized as groups 1 and 2 (Figure 2). Another limitation of priUTR is that it discards those transcripts with less than 3 introns (including the intronless ones) since the transcript do not meet the requirement for partial intron-chain matching. This would limit the number of predicted alternative 3′UTRs. Besides that, priUTR is not applicable to non-model organisms without reference genomes. When priUTR is applied to an organism with a reference genome and poor annotations, caution should be taken as predicted alternative 3′UTRs could be previously unannotated 3′UTR regions.

Thanks to the identification of potential sources of mis-predicted alternative 3′UTRs, several improvements could be made to the priUTR tool in the near future. First, filters could be made to discard genes with multiple overlapped transcripts. Second, filters could be applied to discard those genes with neighbor genes located at 3′ downstream to avoid mis-assembly of transcript. Third, methods could be established to identify alternative 3′UTR transcripts with less than three introns.

Besides the application of priUTR method for characterizing alternative 3′UTRs in sorghum, we attempted to gain insights into the factors associated with the alternative 3′UTRs. Our results strongly indicate an association between m^6^A modification and alternative 3′UTR (Figure 3). Recently, a maize study of RNA m^6^A profiling reveals a clear indication between the m^6^A modification and APA (Luo et al., 2020). There are both similarities and differences between the results of the maize m^6^A study and the results of the present alternative 3′UTR study. In the maize study, genes were split into two sets, one set of genes with single poly(A) site and the other set of genes that contain at least two poly(A) sites and could have APA events. With such an approach for dividing the maize expressed genes, significant associations between potential APA genes and m^6^A modified genes were observed (Luo et al., 2020). The maize study was a genome-wide, large-scale profiling of m^6^A methylated mRNAs without actually determining the mRNAs with alternative 3′UTRs or poly(A) sites. In our sorghum study, the association between APA and m^6^A methylation was discovered the other way around. We first identified the genes using alternative 3′UTRs and then discovered that many of the alternative 3′UTR genes could carry m^6^A methylation. Another feature of our results is that a few hundreds of alternative 3′UTR genes were identified in sorghum, with the potential to carry m^6^A modification. Together with enriched functions, our results could help to narrow down candidate genes for further functional studies with a focus on the cause-and-effect relationship between m^6^A methylation and APA for specific functionally important genes. In addition, our results provide pieces of evidence that alternative 3′UTR usage for some genes (many functionally important) are genotype specific and could be inherent genetically.

For the m^6^A hypothesis, public data from maize studies have been used (Luo et al., 2020; Miao et al., 2020). Maize and sorghum are close relatives, split just 11.9 million years ago (Mya) from a common progenitor (Swigonova et al., 2004). After the divergence, maize experienced allotetraploidization followed by diploidization (Gaut and Doebley, 1997; Xu and Messing, 2008). Therefore, the orthologous relationship between maize and sorghum genes is well established, and many gene regulations remain conserved (Zhang et al., 2017). Such conservation could help to establish an association between RNA m^6^A modification and alternative 3′UTR. For the APA hypothesis, it remains possible that PA machinery proteins, stress responses, or developmental context might be involved in alternative 3′UTRs in sorghum. *Arabidopsis* and rice are plant model species divergent from sorghum. The authentic results of APA or alternative 3′UTRs related to stresses have only been reported by a handful of studies in plants (Sun et al., 2017; Téllez-Robledo et al., 2019). As far as our knowledge is concerned, the target genes of PA machinery proteins have only been studied in *Arabidopsis* (Thomas et al., 2012; Duc et al., 2013; Lin et al., 2017; Téllez-Robledo et al., 2019). Whether or to what extent these APA genes are evolutionarily conserved between *Arabidopsis* and sorghum remains to be determined. Thus, one of the possible explanations for the non-significant results between APA-associated genesets and the sorghum alternative 3′UTR genes could be that the APA genes that respond to stresses or are induced by mutations of PA proteins could be largely unconserved between sorghum and *Arabidopsis*. Overall, our results clearly lead to important testable hypotheses that: (1) m^6^A modifications are functionally related to alternative 3′UTR/APA, yet the cause-and-effect relationship between them needs clarification, and (2) whether the genes with inter-genotype variations in alternative 3′UTR/APA could have functional consequences in sorghum; if yes, what are the consequences in molecular and phenotypic levels.

While conventional RNA-seq studies are prevalent in sorghum, studies on alternative 3′UTR/APA are scarce in non-model plant species. Previously, the full-length transcriptome of sorghum seedling was characterized by PacBio Iso-Seq and identified a few thousand transcripts with APA, demonstrating APA as a common phenomenon (Abdel-Ghany et al., 2016). We acknowledge that the priUTR-based 3′UTR analysis has limitations in the number of alternative 3′UTRs identified and the mis-predictions compared to those specialized pipelines based on the more advanced long-reads sequencing technologies (*i*.*e*., PacBio and Oxford Nanopore) (Abdel-Ghany et al., 2016; Parker et al., 2020). These limitations are partly due to the short-read derived transcriptome assembly and to the limited ability in detecting transcripts with large variations using reference-guided mapping (Stark et al., 2019). Recently, new aspects of mRNA 3′ ends have been revealed to affect post-transcriptional regulation, including poly(A) length and sequence composition (Zhao et al., 2019). This suggests that a long-read-based method to integrate profiles of alternative 3′UTR/APA, poly(A) length and sequence, and m^6^A modifications will be a powerful tool to understand 3′UTR-mediated regulation in the near future.

In summary, our work presents a transcriptome-wide profiling of alternative 3′UTRs in sorghum and identified hundreds of genes as the candidate genes to study the functional effects of alternative 3′UTR usage. The new insights reported here suggest future research directions for 3′UTR genotype specificity and the link between 3′UTR and epi-transcriptome modifications. Additionally, this study exemplifies alternative 3′UTR analysis using conventional RNA-seq, signifying 3′UTR analysis as a valuable addition to routine RNA-seq analysis in plants.

## Data Availability Statement

The RNA-seq data of sorghum used in this study can be found at NCBI Sequence Read Archive (SRA) under accession PRJNA413691. The priUTR program for 3′UTR analysis is available at github (https://github.com/mint1234/3UTR-). All data generated or analyzed during this study are included in this published article and its Supplementary Material.

## Author Contributions

MT and YL designed the study, performed the data analysis, and revised and finalized the manuscript. MT generated the data. YL wrote the manuscript. All authors contributed to the article and approved the submitted version.

## Conflict of Interest

The authors declare that the research was conducted in the absence of any commercial or financial relationships that could be construed as a potential conflict of interest.

## Supplementary Material

Supplementary Figure S1 Schematic diagram of the priUTR algorithm.

Supplementary Figure S2 Distribution of the expression levels for the five groups of predicted alternative 3′UTR genes.

Supplementary Figure S3 Comparison of groups 1, 2, 3, and 5 of sorghum alternative 3′UTR genes with the APA- or m^6^A- associated genesets in *Arabidopsis*, rice, and maize.

Supplementary Figure S4 The distribution of the number of genes overlapped between the public genesets and the randomly selected genes in sorghum by using a permutation test.

Supplementary Figure S5 The distribution of hypergeometric *p*-values between the public genesets and the randomly selected genes in sorghum by using a permutation test.

Supplementary Figure S6 Genome browser views of the 23 genes with genotype-specific alternative 3′UTRs.

Supplementary Table S1 Information about the 612 high-confidence alternative 3′UTR genes.

Supplementary Table S2 Summary information about the public available genesets associated with m^6^A and APA.

Supplementary Table S3 Genesets for m^6^A and APA.

Supplementary Table S4 Hypergeometric tests for the overlap between sorghum alternative 3′UTR genes and the APA- or m^6^A- associated genesets.

Supplementary Table S5 Distribution of the expression levels of the high-confidence alternative 3′UTR genes in Rio, BTx406, and R9188, respectively.

Supplementary Table S6 Functional enrichment results of group 4 genes and the m^6^A-associated 3′UTR subset.

Supplementary Table S7 Information about the 23 genes with genotype-specific alternative 3′UTR.
