# Supplementary material for: Profiling Alternative 3′ Untranslated Regions in Sorghum using RNA-seq Data
Source: Front Genet. 2020 Oct 26;11:556749. doi: 10.3389/fgene.2020.556749 (PMC7649775; doi:10.3389/fgene.2020.556749)
Supplement: Supplementary Figure S1 — Schematic diagram of the priUTR algorithm. [file Data_Sheet_1.PDF]

Profiling Alternative 3' Untranslated Regions in Sorghum using RNA-seq Data

Min Tu[1], Yin Li[1*]

[1]Waksman Institute of Microbiology, Rutgers, The State University of New Jersey, Piscataway, NJ 08854, USA.

*** Correspondence:**
Yin Li
liyin.plant@hotmail.com

Supplementary Material

Figure S1 – Schematic diagram of the priUTR algorithm.

Figure S2 – Distribution of the expression levels for the five groups of predicted alternative-3'UTR genes.

Figure S3 – Comparison of the Groups 1, 2, 3, and 5 of sorghum alternative-3'UTR genes with the APA- or m^{6}A- associated genesets in Arabidopsis, rice and maize.

Figure S4 – The distribution of number of genes overlapped between the public genesets and the randomly selected genes in sorghum by using permutation test.

Figure S5 – The distribution of hypergeometric p-values between the public genesets and the randomly selected genes in sorghum by using permutation test.

Figure S6 –Genome browser views of the 23 genes with genotype specific alternative 3'UTRs.

Table S1 – Information about the 612 high-confident alternative-3'UTR genes.

Table S2 – Summary information about the public available genesets associated with m^{6}A and APA.

Table S3 – Genesets for m^{6}A and APA.

Table S4 – Hypergeometric tests for the overlapping between sorghum alternative-3'UTR genes and the APA- or m^{6}A- associated genesets.

Table S5 – Distribution of the expression levels of the high-confident alternative-3'UTR genes in Rio, BTx406, and R9188, respectively.

Table S6 – Functional enrichment results of Group 4 genes and the m^{6}A-associated 3'UTR subset.

Table S7 – Information about the 23 genes with genotype specific alternative 3'UTR.

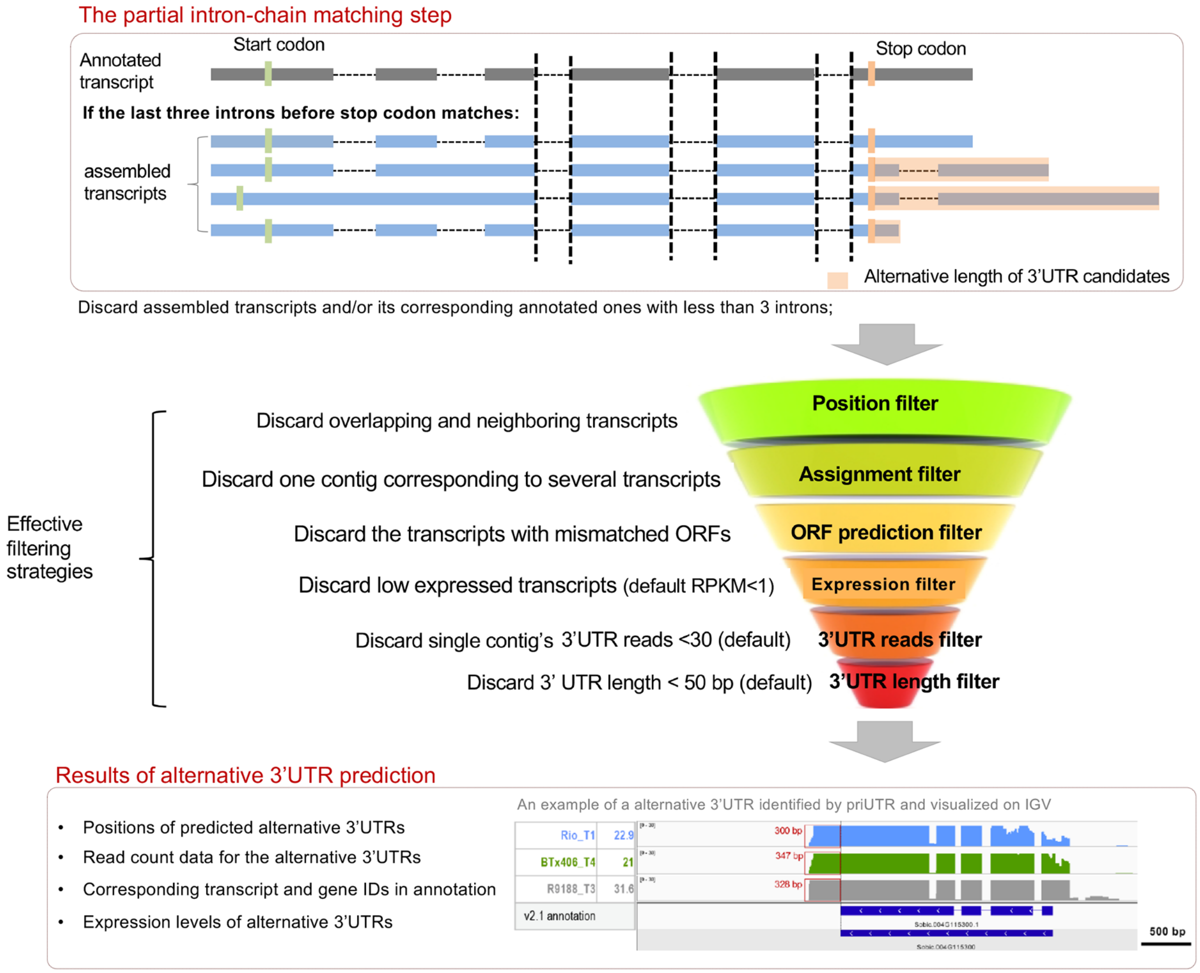

Figure S1. Schematic diagram shows the algorithm of priUTR method and the filters for identifying alternative 3’UTRs from RNA-seq data.

The priUTR program belongs to the type 2 bioinformatic tools for identifying alternative 3’UTRs or poly(A) sites according to a comprehensive review by Chen et al., 2020. It takes the output files (i.e., transcriptome assembly GTF files) of classic RNA-seq analysis pipelines as the input. Classic RNA-seq analysis pipelines (*e.g.*, Tophat-Cufflinks (Trapnell et al., 2012) and HISAT2-StringTie (Pertea et al., 2016)) rely on a reference genome and annotation to perform splice junction-aware read alignment and can assemble transcriptome with the guide of the reference. The priUTR program compares reconstructed transcripts (in our case, Tophat-Cufflinks was used) with their corresponding annotated transcripts by a partial intron-chain matching algorithm, which matches three consecutive introns 5’upstream of the stop codon. After the relationships between reconstructed transcripts and annotated transcripts were established, a series of filters were applied in the priUTR program to improve its prediction accuracy: (1) discard the transcripts without intron or with less than three introns (at the intron-chain matching step); (2) discard the transcripts corresponding to multiple or overlapping annotated transcripts; (3) discard the transcripts with mismatched stop codon; (4) discard the transcripts with low expression levels (RPKM < 1); (4) discard the transcripts with less than 30 reads mapped to the predicted alternative 3’UTRs. After filtering, priUTR outputs the details about putative alternative 3’UTRs, including their corresponding transcripts and gene models, their corresponding annotated 3’UTRs, length of the alternative and annotated 3’UTRs, genomic positions of the alternative 3’UTRs,

number of reads mapped to the alternative 3’UTRs and expression of 3’UTRs (in RPKM). More detailed information is available at: https://github.com/mint1234/3UTR-.

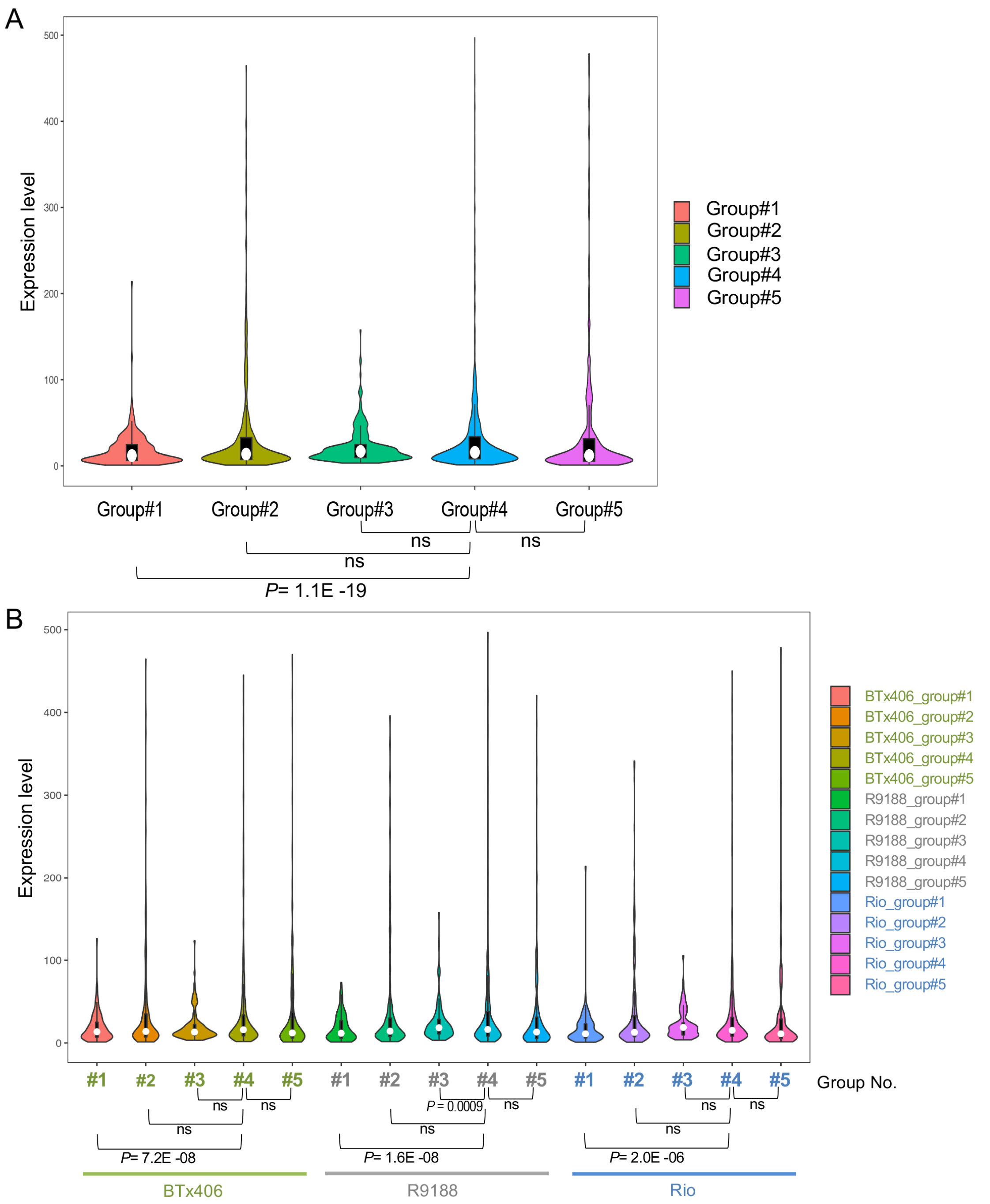

Figure S2. Distribution of the expression levels for the five groups of genes with predicted alternative 3’UTRs. (A) Gene expression levels for Groups 1, 2, 3, 4 and 5 pooled for all of the three sorghum genotypes were visualized using violin plot and pairwise compared by Welch two-sample *t*-test (P<0.05). (B) Genes expression levels for Groups 1, 2, 3, 4, and 5 for BTx406, R9188, and Rio,

3

respectively, were visualized using violin plot and pairwise compared within each genotype (Welch two-sample *t*-test, P<0.05).

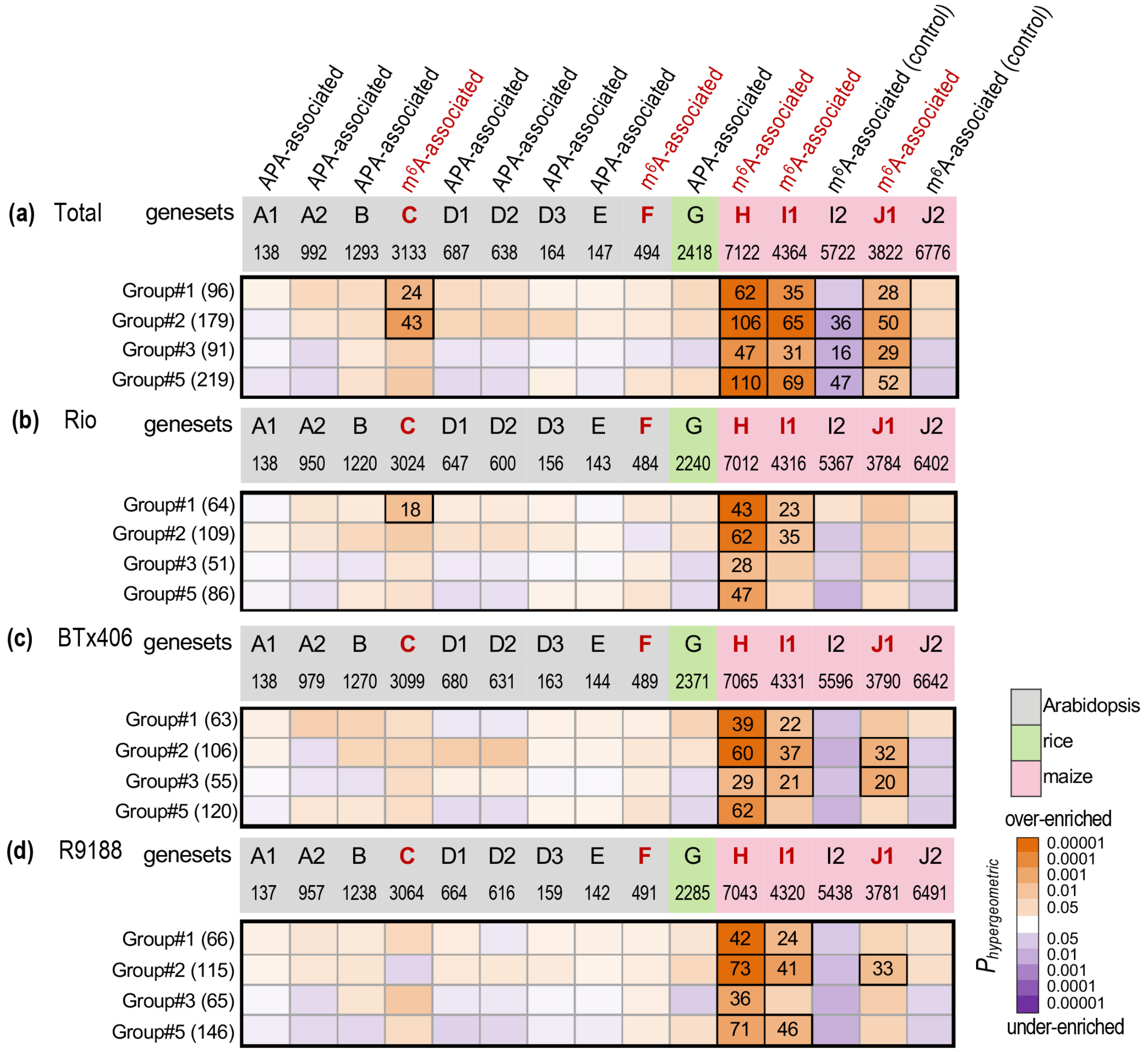

Figure S3. Significant overlapping between alternative-3’UTR groups (Groups 1, 2, 3 and 5) with m^{6}A-associated genesets were detected for all three genotypes and for each of the three sorghum genotypes. The significance of geneset overlapping was determined by hypergeometric test (P<0.01) for the groups pooled from all three genotypes (**a**), and for the groups in Rio (**b**), BTx406 (**c**) and R9188 (**d**), respectively. The significant overlaps between genesets are labeled in black boxes and the number of overlapping genes is shown. Information for the published genesets used in this analysis was available in Figure 3A and Supplementary Table S2. The number of genes expressed in each sorghum genotype are listed below the geneset names (*i.e.*, A1, A2, B, C, D1, D2, D3, E, F, G, H, I1, I2, J1, and J2).

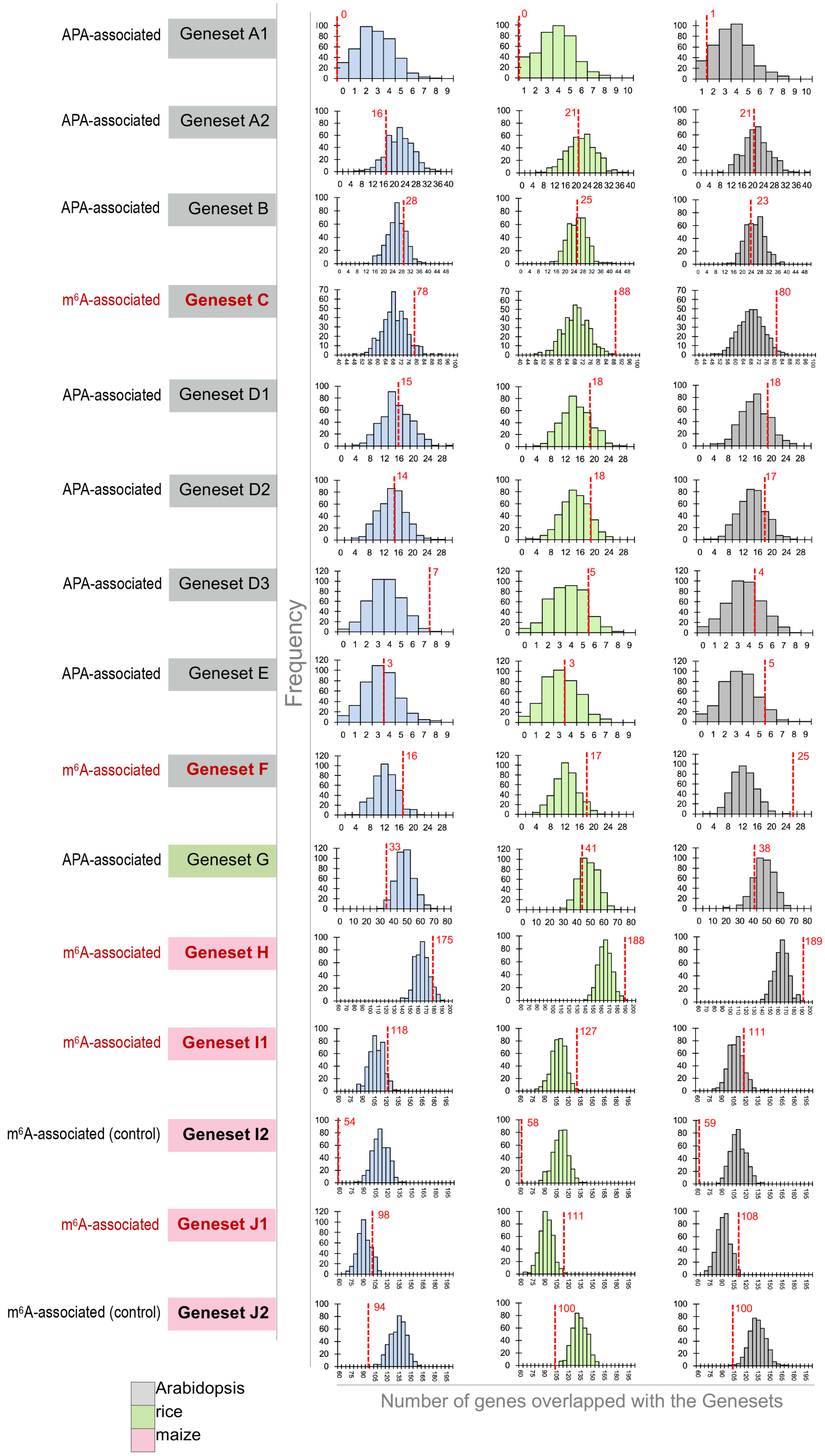
APA-associated
Geneset A1
APA-associated
Geneset A2
APA-associated
Geneset B
m6A-associated
Geneset C
APA-associated
Geneset D1
APA-associated
Geneset D2
APA-associated
Geneset D3
APA-associated
Geneset E
m6A-associated
Geneset F
APA-associated
Geneset G
m6A-associated
Geneset H
m6A-associated
Geneset I1
m6A-associated (control)
Geneset I2
m6A-associated
Geneset J1
m6A-associated (control)
Geneset J2
Frequency
Number of genes overlapped with the Genesets
Arabidopsis
rice
maize

5

Figure S4. The distribution of number of genes overlapped between several public genesets (*i.e.*, A1, A2, B, C, D1, D2, D3, E, F, G, H, I1, I2, J1, and J2) and randomly selected expressed genes in Rio, BTx406, and R9188, respectively, were estimated using 400-times permutation test and are shown in histogram. The results for Rio, BTx406, and R9188 are shown in blue, green, and grey histograms, respectively. The red dotted lines label the observed number of genes overlapped between the Group-4 alternative-3'UTR genes and the genesets A1, A2, B, C, D1, D2, D3, E, F, G, H, I1, I2, J1, and J2. Color shades on the geneset names indicate the original species.

6

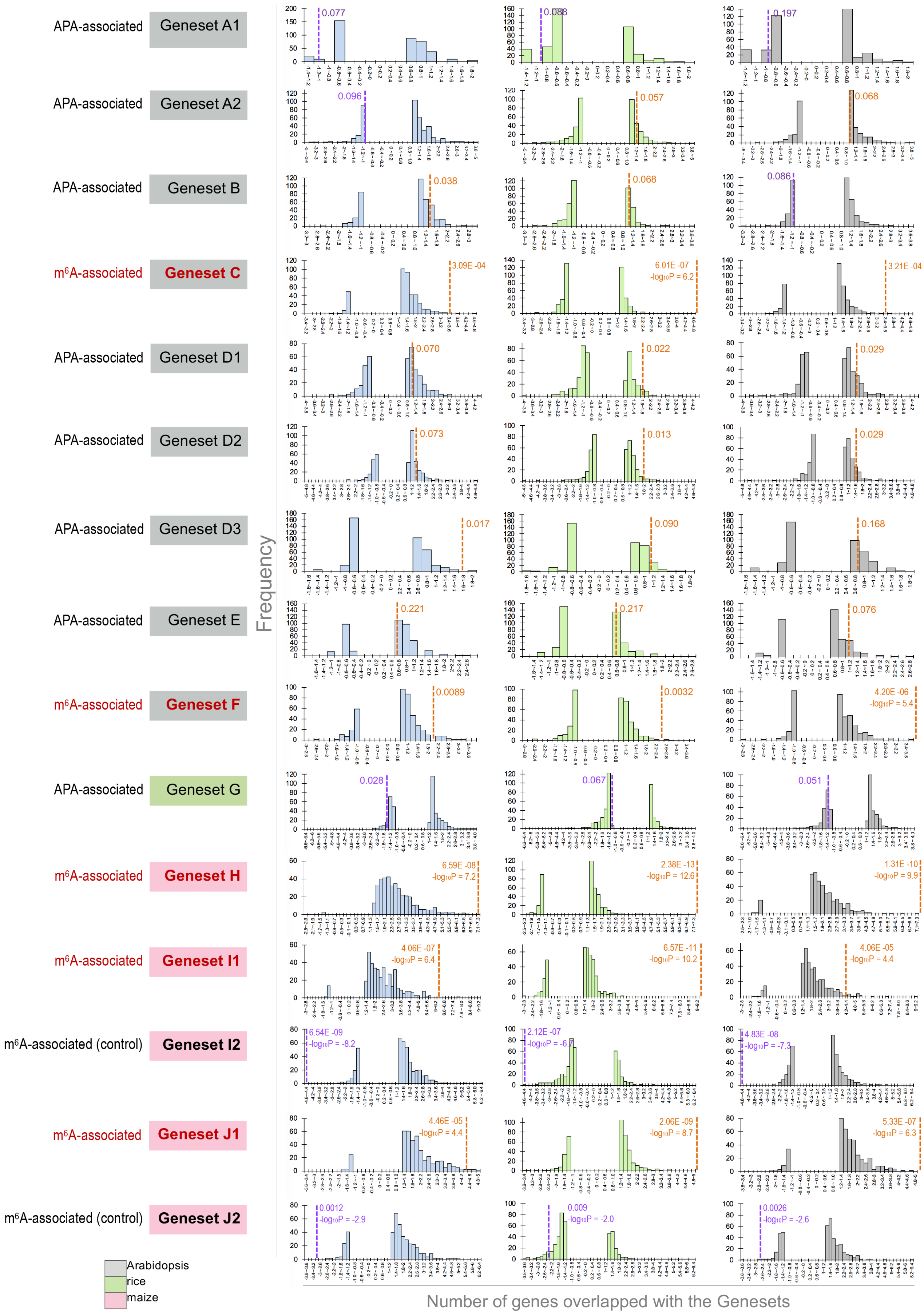
APA-associated
Geneset A1
0.077
0.088
0.197
APA-associated
Geneset A2
0.096
0.057
0.068
APA-associated
Geneset B
0.038
0.068
0.086
m6A-associated
Geneset C
3.09E -04
6.01E -07
-log10P = 6.2
3.21E -04
APA-associated
Geneset D1
0.070
0.022
0.029
APA-associated
Geneset D2
0.073
0.013
0.029
APA-associated
Geneset D3
0.017
0.090
0.168
APA-associated
Geneset E
0.221
0.217
0.076
m6A-associated
Geneset F
0.0089
0.0032
4.20E -06
-log10P = 5.4
APA-associated
Geneset G
0.028
0.067
0.051
m6A-associated
Geneset H
6.59E -08
-log10P = 7.2
2.38E -13
-log10P = 12.6
1.31E -10
-log10P = 9.9
m6A-associated
Geneset I1
4.06E -07
-log10P = 6.4
6.57E -11
-log10P = 10.2
4.06E -05
-log10P = 4.4
m6A-associated (control)
Geneset I2
6.54E -09
-log10P = -8.2
2.12E -07
-log10P = -6.7
4.83E -08
-log10P = -7.3
m6A-associated
Geneset J1
4.46E -05
-log10P = 4.4
2.06E -09
-log10P = 8.7
5.33E -07
-log10P = 6.3
m6A-associated (control)
Geneset J2
0.0012
-log10P = -2.9
0.009
-log10P = -2.0
0.0026
-log10P = -2.6
Frequency
Number of genes overlapped with the Genesets
Arabidopsis
rice
maize

Figure S5. The distribution of hypergeometric p-values between several public genesets (*i.e.*, A1, A2, B, C, D1, D2, D3, E, F, G, H, I1, I2, J1, and J2) and randomly selected expressed genes in Rio, BTx406, and R9188, respectively, were estimated using 400-times permutation test and are shown in histogram. The results for Rio, BTx406, and R9188 are shown in blue, green, and grey histograms, respectively. A p-value above zero means over-enrichment of a public geneset in the randomly selected sorghum genes; while a p value below zero means under-enrichment of a public geneset in the randomly selected sorghum genes. The observed hypergeometric p values between the Group-4 alternative-3’UTR genes and the genesets A1, A2, B, C, D1, D2, D3, E, F, G, H, I1, I2, J1, and J2, respectively, are labeled, with orange standing for over-enrichment, purple for under-enrichment. Color shades on the geneset names indicate the original species.

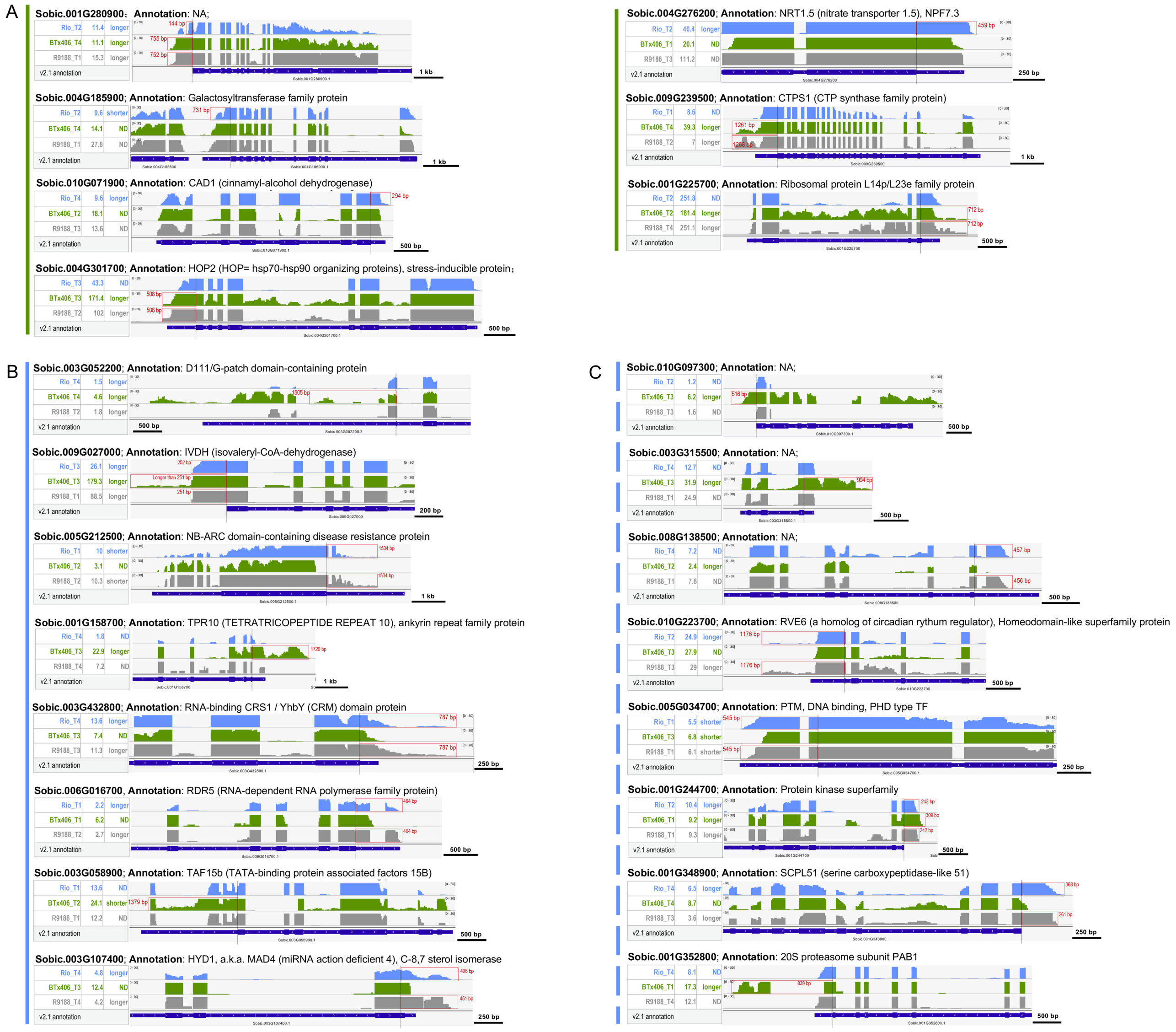

Figure S6. Genome browser views of the 23 genes with genotype specific alternative 3’UTR patterns. Based on the SNPs identified using RNA-seq reads from Rio, BTx406 and R9188, the genes in R9188 were identified to be originated from Rio or introgressed from BTx406. (**A**) The BTx406-introgressed R9188 alleles showing the same alternative 3’UTR with those in BTx406 but differed from those in

Rio. (**B**) The Rio-originated R9188 alleles showing the same alternative 3'UTR with those in Rio but differed from those in BTx406. (**C**) The R9188 alleles that have the same alternative 3'UTR with those in Rio but differ from those in BTx406, and lacks SNPs for introgression regions.

9
